# Higher whole‐body and exogenous postprandial glucose oxidation in the luteal versus follicular phase in healthy females

**DOI:** 10.14814/phy2.70876

**Published:** 2026-06-17

**Authors:** Alexa Govette, Daniel W. D. West, Madeleine Pettit, Eric Antonen, Anessa Koussiouris, Daniel R. Moore, Jenna B. Gillen

**Affiliations:** ^1^ Faculty of Kinesiology and Physical Education University of Toronto Toronto Ontario Canada; ^2^ KITE, Toronto Rehabilitation Institute, University Health Network Toronto Ontario Canada

**Keywords:** blood glucose, carbohydrate oxidation, estradiol, fat oxidation, substrate utilization

## Abstract

Menstrual cycle phase can influence indices of carbohydrate metabolism at rest and during exercise; however, limited research has explored whether there are menstrual phase‐specific differences in substrate metabolism in response to oral feeding. Using a within‐subjects crossover design, 15 eumenorrheic females completed a metabolic trial in the follicular phase (FP; day 7 ± 2) and the luteal phase (LP; day 22 ± 3; 8 ± 2 days post‐ovulation) of a single menstrual cycle. Following an overnight fast, participants consumed a 75 g glucose beverage enriched with a [U‐^13^C_6_] D‐glucose tracer. Capillary glucose concentrations, exogenous glucose oxidation, expired carbon dioxide percentage (L%CO_2_), and whole‐body carbohydrate and fat oxidation rates were measured fasted and across 3‐h postprandially. Peak 3‐h glucose concentration was higher in LP versus FP (*p* = 0.04), with no differences in 3‐h mean glucose or incremental area under the curve between phases (*p* = 0.34–0.63). Whole‐body (*p* = 0.03) and exogenous carbohydrate oxidation (*p* = 0.002) were higher in LP versus FP. %LCO_2_ was elevated above fasting at all postprandial timepoints (*p* < 0.001) with no differences between phases (*p* = 0.84). In conclusion, following an oral glucose load, peak blood glucose concentration and carbohydrate oxidation rates were elevated in LP versus FP. These findings suggest postprandial substrate utilization differs by menstrual cycle phase in healthy females.

## 1 INTRODUCTION

The female menstrual cycle can be divided into pre‐ and post‐ovulatory phases, commonly referred to as the follicular phase (FP) and luteal phase (LP). During the majority of the FP, 17‐β estradiol (estradiol) and progesterone concentrations are low, whereas both hormones rise and remain elevated in the LP (Isacco et al., 2012; Wismann & Willoughby, 2006). These fluctuations in sex hormone concentrations across the menstrual cycle have been demonstrated to influence various metabolic and respiratory responses in females (Behan & Wenninger, 2008; Schieren et al., 2024; Schmalenberger et al., 2019). As such, comparing physiological and health‐related outcomes across the menstrual cycle is essential for understanding potential phase‐specific differences that may influence disease risk and well‐being in females.

Elevated progesterone in the LP has been associated with increased insulin resistance (Schieren et al., 2024). For example, several studies using fasting indices such as the homeostasis model of insulin resistance have demonstrated greater insulin resistance in the LP compared to the FP in healthy, eumenorrheic females (Yeung et al., 2010; Zarei et al., 2013). Additionally, findings from intravenous glucose tolerance tests further support this pattern, showing reduced insulin sensitivity in the LP compared to FP among two small cohorts (*n* = 8–12) of healthy eumenorrheic females (Pulido & Salazar, 1999; Valdes & Elkind‐Hirsch, 1991). However, intravenous and fasting measures of insulin resistance do not reflect the blood glucose response to oral feeding, which more closely mirrors the body's typical nutrient exposure across the menstrual cycle. Considering that insulin resistance is associated with elevations in blood glucose concentration, particularly following meals (Wysham & Shubrook, 2020), pre‐menopausal women may be particularly susceptible to postprandial hyperglycemia in the LP of the menstrual cycle.

Few studies have examined the blood glucose responses to oral feeding across the menstrual cycle using oral glucose tolerance tests (OGTT) (Bonora et al., 1987; Brennan et al., 2009; Cudworth & Veevers, 1975). Brennan et al. (2009) observed higher mean blood glucose during the 90 min following a 50 g OGTT in the LP compared to the FP among a small cohort (*n* = 9) of healthy eumenorrheic females. In contrast, Bonora et al. (1987) observed no difference in mean blood glucose over 120 min following a 75 g OGTT in the LP versus FP in non‐obese eumenorrheic females; however, this was a between‐subject design which may have increased variability. Using a within‐subject design among 22 eumenorrheic females, Cudworth and Veevers (1975) came to similar conclusions in that there was no difference in mean blood glucose between the FP and LP in response to a 100 g OGTT. Discrepancies among studies may be attributed to differences in the glucose dose (50–100 g), measurement period (90–120 min postprandial) and/or the method for menstrual cycle phase determination. These studies relied solely on calendar‐based counting to determine participants' testing days, without any measurement to confirm ovulation. This approach is imprecise and susceptible to error due to the interindividual variability in menstrual cycle patterns among women (Elliott‐Sale et al., 2025). Given these methodological limitations, further research is needed to understand whether menstrual cycle phase impacts postprandial glycemia.

In addition to blood glucose responses, substrate oxidation in response to feeding may also differ across the menstrual cycle. Following glucose ingestion, the body increases carbohydrate oxidation as a result of increased availability, although it is currently unknown whether menstrual cycle phase modulates this response. During aerobic exercise, elevations in estradiol through exogenous supplementation (Hamadeh et al., 2005) or as seen in the LP versus early FP (Devries et al., 2006; Hackney, 1999; Zderic et al., 2001) result in decreased carbohydrate oxidation as measured via indirect calorimetry. However, whether menstrual cycle phase‐based differences in substrate oxidation exist in the rested postprandial state remains unclear.

The purpose of the present study was to investigate the effects of menstrual cycle phase (FP vs. LP) on postprandial glycemic control and substrate oxidation at rest and in response to an oral glucose load in pre‐menopausal naturally cycling females. We hypothesized that postprandial capillary glucose concentrations would be higher during LP compared to FP due to previous reports of decreased insulin sensitivity during LP. Additionally, based on the available evidence during exercise, we hypothesized that resting and postprandial carbohydrate oxidation would be lower in LP compared to FP.

## 2 MATERIALS AND METHODS

### 2.1 Participants and ethics approval

Fifteen healthy females between 18 and 35 years old were recruited from the Greater Toronto Area via poster and web‐based advertisement from July 2022 to April 2023. All participants were eumenorrheic, defined by a self‐reported menstrual cycle length between 21 and 35 days for the 3 months prior to study enrollment (Elliott‐Sale et al., 2021), not using any hormonal contraceptive medication within the past 6 months, a body mass index (BMI) of ≥18.5 kg/m^2^ and ≤ 27 kg/m^2^, and not following any specialized diets (e.g., ketogenic diet and intermittent fasting). Females were excluded from the study if they reported a diagnosed medical condition under the care of a physician, were pregnant (currently or within the last year), or reported regular tobacco and/or drug use.

Sample size estimates were determined using G*Power 3.1 based on previously reported effect sizes (Cohen's *f* = 0.35) for the difference in postprandial glucose area under the curve (AUC; primary outcome) across time in FP compared to LP (Cudworth & Veevers, 1975). To detect a significant effect (*p* < 0.05) between conditions if one exists with 80% power, a sample size of *n* = 12 was required. To accommodate potential attrition, we recruited *n* = 15 participants.

This study was conducted according to the guidelines laid down in the Declaration of Helsinki and all procedures involving human participants were approved by the University of Toronto Health Sciences Research Ethics Board (#40004). Written informed consent was obtained from all participants.

### 2.2 Baseline testing and pre‐trial preparation

Following a 12‐h overnight fast, body mass and composition were assessed in the laboratory using air displacement plethysmography (BodPod; Cosmed USA, Inc., Concord, CA) and waist circumference was measured using a retractable tape measure (Model number: B0BTKVKYDN; ExeQianming Inc., Chinath). Participants wore an accelerometer (Actigraph; GT3X‐BT, Pensacola, FL) to monitor their physical activity for 3 days prior to both metabolic trials in order to characterize habitual physical activity and step count. Participants were also instructed to refrain from structured physical activity (verified via accelerometry), alcohol consumption, and replicate their diet 24 h before each trial to minimize the effects of acute exercise and dietary changes on substrate oxidation. Participants completed a 24‐h dietary record prior to their first metabolic trial using a de‐identified MyFitnessPal study account. Prior to the second trial, participants were provided a copy of their recorded dietary intake and were instructed to replicate this intake during the 24‐h period preceding the trial. All dietary records were checked for completion by a study investigator. Participants were also provided with a Lumen device (Metaflow Ltd., Tel Aviv, Israel), described further below, with study‐specific login details and instructions for measuring expired breath %CO_2_ (L%CO_2_). A 3‐day run‐in period was included to minimize learning effects and ensure accurate synchronization of the Lumen software to individual daily breath responses through participant familiarization with the device, as we have reported previously (Govette & Gillen, 2024).

### 2.3 Metabolic trial overview

**FIGURE 1:**
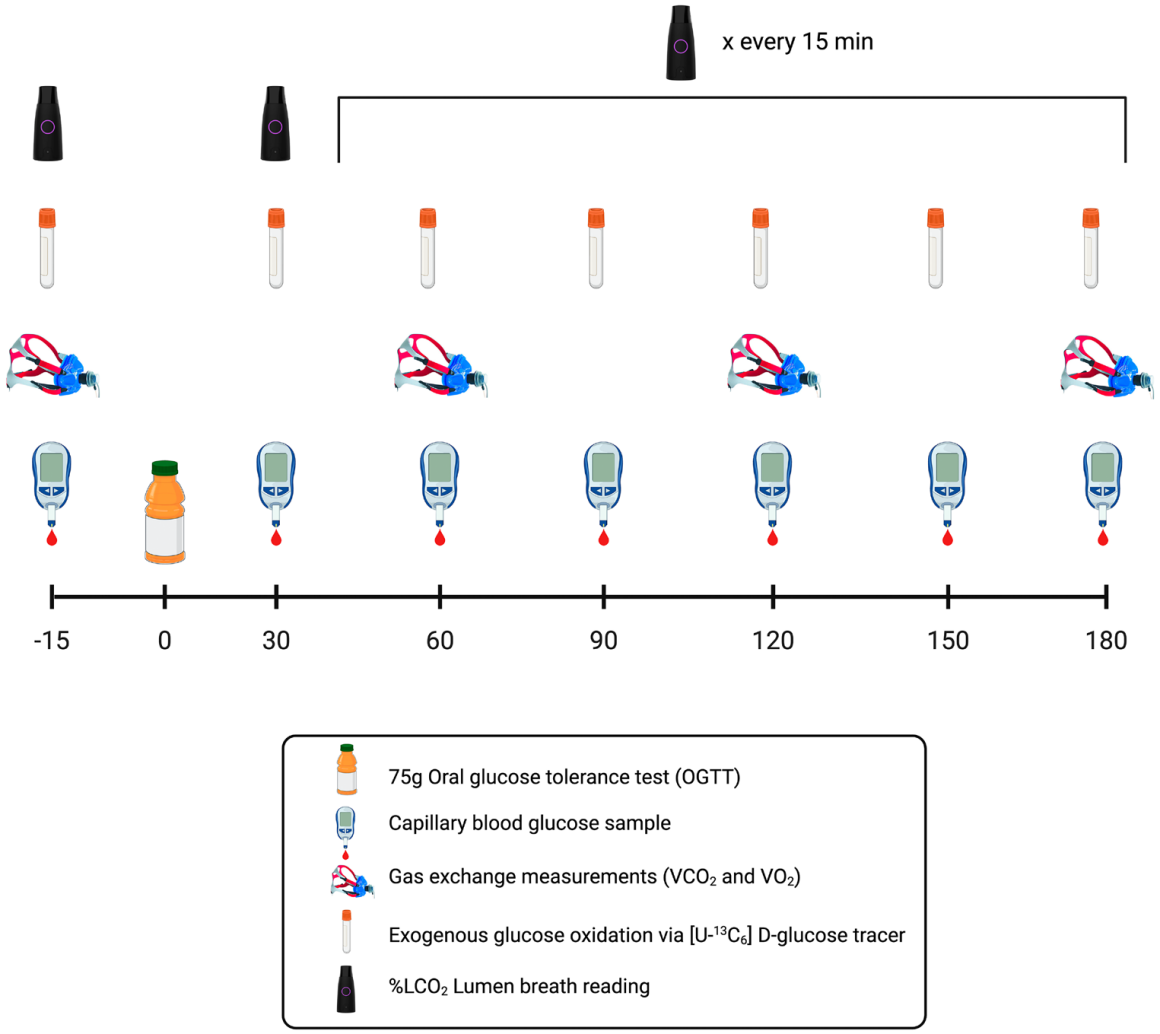
Schematic representation of experimental trials. Created in https://BioRender.com.

Participants underwent two 3.5 h metabolic trials in the FP and LP of a single menstrual cycle. The order of the metabolic trials was not randomized in order to directly compare substrate metabolism across menstrual cycle phases within a single menstrual cycle (FP followed by LP). For each metabolic trial, participants arrived at the laboratory following a 12‐h overnight fast. Upon arrival, participants remained seated for 20 min prior to obtaining fasted measurements. Next, participants ingested a 75 g glucose beverage (NERL™, ThermoFisher, CAN) enriched with 75 mg (0.1%) of stable isotope tracer (D‐glucose U‐^13^C_6_, Cambridge Isotope Laboratories). Participants remained seated in the laboratory for 3 h following ingestion of the test beverage, and measurements of postprandial substrate metabolism were collected throughout. A schematic representation of the metabolic trials is presented in Figure 1.

### 2.4 Menstrual cycle phase determination

Menstrual cycle phase was determined using a combination of calendar‐based tracking and at‐home urinary ovulation prediction tests (Easy@Home). Participants completed a short questionnaire to report the start date of menses for their three most recent menstrual cycles, which was used to calculate average cycle length and estimate timing for metabolic trials. The follicular phase (FP) metabolic trial was conducted between days 3 and 10 of their menstrual cycle, based on self‐reported onset of menses. A urinary ovulation test was performed in the laboratory the morning of FP to confirm that participants were not ovulating. During this menstrual cycle, participants also conducted daily ovulation tests at home starting on day 8 and notified a study investigator when they received a positive test (verified via photo documentation). The luteal phase (LP) trial was scheduled 4–9 days following a positive ovulation test, resulting in LP trial days that ranged from day 20 to 26 of the menstrual cycle.

### 2.5 Analytical procedures

#### 2.5.1 Capillary glucose measurement

Capillary glucose concentration was determined at baseline (fasted) and every 30 min for 3 h following ingestion of the 75 g glucose beverage. Capillary blood was obtained via a finger prick using a lancet and applied to a test strip, which was then analyzed using a glucose meter (ContourNext). At each time point, capillary glucose concentration was measured in duplicate and averaged. In the event that the glucose readings differed by >10%, a third measurement was obtained, and the two most consistent readings were averaged. Capillary blood glucose concentration at each time point was calculated and plotted across time, and 3 h postprandial glucose mean, peak, and incremental area under the curve (iAUC) using the trapezoidal rule were determined.

#### 2.5.2 Indirect calorimetry

Gas exchange variables, including volume of oxygen consumed per minute (VO_2_, L/min), volume of carbon dioxide expired per minute (VCO_2_, L/min), fraction of expired oxygen (FeO_2_, %) and carbon dioxide (FeCO_2_, %), breathing frequency (Rf, breaths/min), tidal volume (VT, L), and minute ventilation of gas exhaled (VE, L/min) were measured via indirect calorimetry at fasted baseline and once every hour for 3 h following ingestion of the 75 g glucose beverage. All measurements were collected with participants in a seated upright position with instructions to breathe normally into a silicone face mask (Hans Rudolph7450 Series V2 Mask) connected to a metabolic cart (ParvoMedics TrueOne 2400). At each data collection timepoint, gas exchange variables were recorded as 30‐s averages for 15 min. The first 5 min of each measurement period was discarded, resulting in a 10‐min period that was used for analysis. Respiratory exchange ratio (RER) was calculated as the ratio of VCO_2_ to VO_2_. Rates of total non‐protein carbohydrate and fat oxidation were calculated from VO_2_ and VCO_2_ values using the following equations, where any negative values were assumed as zero oxidation for that substrate (Jeukendrup et al., 1995; Péronnet & Massicotte, 1991):

$$\text{Carbohydrate oxidation}\;\left(g/\min\right):4.585\times {\mathrm{VCO}}_{2}–3.226\times {\mathrm{VO}}_{2}$$

$$\mathrm{Fat}\;\text{oxidation}\;\left(g/\min\right):1.695\times {\mathrm{VO}}_{2}–1.701\times {\mathrm{VCO}}_{2}$$

All gas exchange variables were calculated at each time interval (fasted, 60, 120, and 180 min postprandial).

#### 2.5.3 Exogenous glucose oxidation via [U‐ ^13^C_6_ ] D‐glucose tracer

Breath samples were collected in 10 mL additive‐free evacuated tubes (BD, Franklin Lakes, NJ) using a collection mechanism that permits removal of dead air space (Single use collection bags, Easy Sampler System, QuinTron, Terumo Medical) at fasted baseline, and every 30 min for 3 h following oral ingestion of the 75 g glucose beverage. Samples were stored at room temperature until the subsequent determination of ^13^CO_2_ enrichment by isotope ratio mass spectrometry (ID Microbreath, Compact Science Systems, UK). Atom percent excess (APE) ^13^C was calculated from delta PDB values as follows:

$${\mathrm{APE}}^{13}C=100/\left(1/\left(\left(\text{delta}/1000+1\right)\times R_{\mathrm{ref}}\right)+1\right)$$

where R ref. = 0.0112372 for PDB.

Exogenous glucose oxidation (Exo Ox) was calculated as previously described (Jeukendrup et al., 1999; Mosora et al., 1976).

$$\mathrm{Exo}\;\mathrm{Ox}=\frac{\mathrm{EC}O_{2}}{E_{\mathrm{ing}}}\times \dot{V}CO_{2}\times \frac{1}{k_{1}}\times \frac{1}{k_{2}}$$

where ECO_2_ is the enrichment of ^13^CO_2_ measured in breath samples (APE; sample minus fasted baseline), *E*_ing_ is the enrichment of the glucose drink that was ingested (atom percent excess), $\dot{V}{\mathrm{CO}}_{2}$ is the rate of carbon dioxide production (in L/h) determined via indirect calorimetry. Indirect calorimetry was performed at hourly time points; for calculation of exogenous glucose oxidation at intermediate sampling times (30, 90, and 150 min), the proceeding closest measured VCO_2_ value was used (i.e., 60 min for 30 min, 120 min for 90 min, and 180 min for 150 min). *k*_1_ is a correcting factor equal to 0.8 for the incomplete recovery of ^13^CO_2_ in breath (Robert et al., 1987) and *k*_*2*_ is equal to 0.7467 (volume of CO_2_ in liters produced by the oxidation of 1 g glucose).

#### 2.5.4 L%CO_2_ via lumen

The Lumen device uses a single breath measure involving a deep inhalation, a 10‐s breath hold and a flow‐rate controlled exhalation procedure to quantify expired %CO_2_. Based on the assumption that oxygen consumption is constant under resting conditions, changes in expired CO_2_ reflect shifts in whole‐body fuel utilization whereby a lower %CO_2_ is reflective of greater fat oxidation and a higher %CO_2_ indicates greater carbohydrate oxidation (Melzer, 2011). Previous laboratory‐based studies have compared the L%CO_2_ with indirect calorimetry‐derived respiratory exchange ratio (RER) values in both the fasted and postprandial states and demonstrated that a 0.28% increase in L%CO_2_ corresponded to a 0.09 increase in RER (Lorenz et al., 2020; Roberts et al., 2023). Our laboratory has previously used L%CO_2_ as an indirect measure of fat oxidation following exercise (Govette & Gillen, 2024). In the present study, Lumen was used to assess substrate oxidation at rest following an overnight fast, and every 15 min for 3 h following oral ingestion of the 75 g glucose beverage. At each time point, two breath maneuvers were performed for measurement of L%CO_2_, which was automatically stored to an online database through the Lumen smartphone application. The %CO_2_ for each breath measurement was determined through two replicate measures with an acceptable difference <0.2%. If the error margin was >0.2%, a third measurement was taken and the two breaths with the smallest difference were considered. L%CO_2_ breath measurements were calculated and plotted across time.

### 2.6 Statistical analyses

All statistical analyses were completed using IBM SPSS Statistics (Version 29.0.1.0), and all graphs were created in GraphPad Prism (GraphPad Software, San Diego, Calif., USA). Dependent variables with a time component, including capillary blood glucose concentration, measures of gas exchange variables (VO_2_, VCO_2_, RER, VT, VE, Rf, FeO_2_, and FeCO_2_), rates of carbohydrate and fat oxidation calculated from measurements collected via indirect calorimetry, exogenous glucose oxidation and %LCO_2_ were analyzed using a two‐factor (phase × time) repeated measures analysis of variance (ANOVA). In the event an ANOVA yielded a significant interaction or main effect, pairwise comparisons were determined post hoc using Bonferroni corrected *t*‐tests. We also determined menstrual cycle phase differences in fasting and 3 h postprandial mean for each of the aforementioned gas exchanged variables, respectively, using a paired students *t*‐test. Differences in 3 h postprandial capillary glucose mean, peak, and iAUC between phases and 3 h mean, and total grams of glucose oxidized for exogenous postprandial glucose oxidation were analyzed using a paired students *t*‐test. All data are reported as the mean ± standard deviation (SD), and statistical significance was set as *p* < 0.05. Partial eta‐squared ($\eta_{p}^{2}$) values were calculated to estimate the effect sizes (small: 0.01, medium: 0.06, and large: 0.14) for main effects and interactions where necessary. Cohen's *d* were calculated to estimate effect size (small: 0.2, medium: 0.5, large: 0.8, and very large: 1.3) for *t*‐tests and post‐hoc comparisons. Although gas exchange data were collected for all 15 participants, *n* = 1 was excluded from the analysis due to technical issues with data collection. Therefore, the data presented for all gas exchange variables measured via indirect calorimetry are presented as *n* = 14. For exogenous glucose oxidation, *n* = 13 participants are included in the analysis. Two participants were excluded due to undetectable tracer enrichment in breath samples (*n* = 1) and data points that exceeded 3 standard deviations above the mean (*n* = 1).

## 3 RESULTS

### 3.1 Participant characteristics and pre‐trial controls

Participant characteristics are summarized in Table 1. Participants completed their FP and LP metabolic trials on day 7 ± 2 (range: day 4–10) and 22 ± 3 (range: day 17–27; 8 ± 2 days post‐ovulation) of a single menstrual cycle, respectively. There were no differences in habitual step count (FP: 9072 ± 3470 vs. LP: 9991 ± 4828 steps/day, *p* = 0.79, *d* = 0.06) or minutes of moderate‐to‐vigorous physical activity (FP: 64 ± 28 vs. LP: 65 ± 32 min/day, *p* = 0.96, *d* = 0.01) in the 3‐day monitoring period before metabolic trials. There was also no difference between FP and LP in self‐reported energy (FP: 1675 ± 428 vs. LP: 1638 ± 582 kcal, *p* = 0.82, *d* = 0.07), carbohydrate (FP: 191 ± 38 vs. LP: 179 ± 73 g, *p* = 0.53, *d* = 0.21), fat (FP: 66 ± 29 vs. LP: 64 ± 31 g, *p* = 0.76, *d* = 0.07), or protein (FP: 83 ± 34 vs. LP: 85 ± 33 g, *p* = 0.86, *d* = 0.05) intake 24‐h before trials.

**TABLE 1:** Participant characteristics.

Variable (*n* = 15)	
Age (year)	25 ± 5
Anthropometrics	
Height (cm)	165 ± 5
Weight (kg)	60 ± 8
BMI (kg/m^2^)	22 ± 3
Waist circumference (cm)	74 ± 6
Fat mass (%)	25.9 ± 7.1
Fat mass (kg)	15.9 ± 5.7
Fat‐free mass (%)	74.1 ± 7.1
Fat‐free mass (kg)	44.5 ± 5.2
Menstrual cycle	
Cycle length (days)	30 ± 3
Ovulation (day)	14 ± 2
FP (day)	7 ± 2
LP (day)	22 ± 3

*Note*: Values are expressed as means ± SD.

Abbreviations: BMI, body mass index; FP, follicular phase trial; LP, luteal phase trial.

### 3.2 Postprandial blood glucose

**FIGURE 2:**
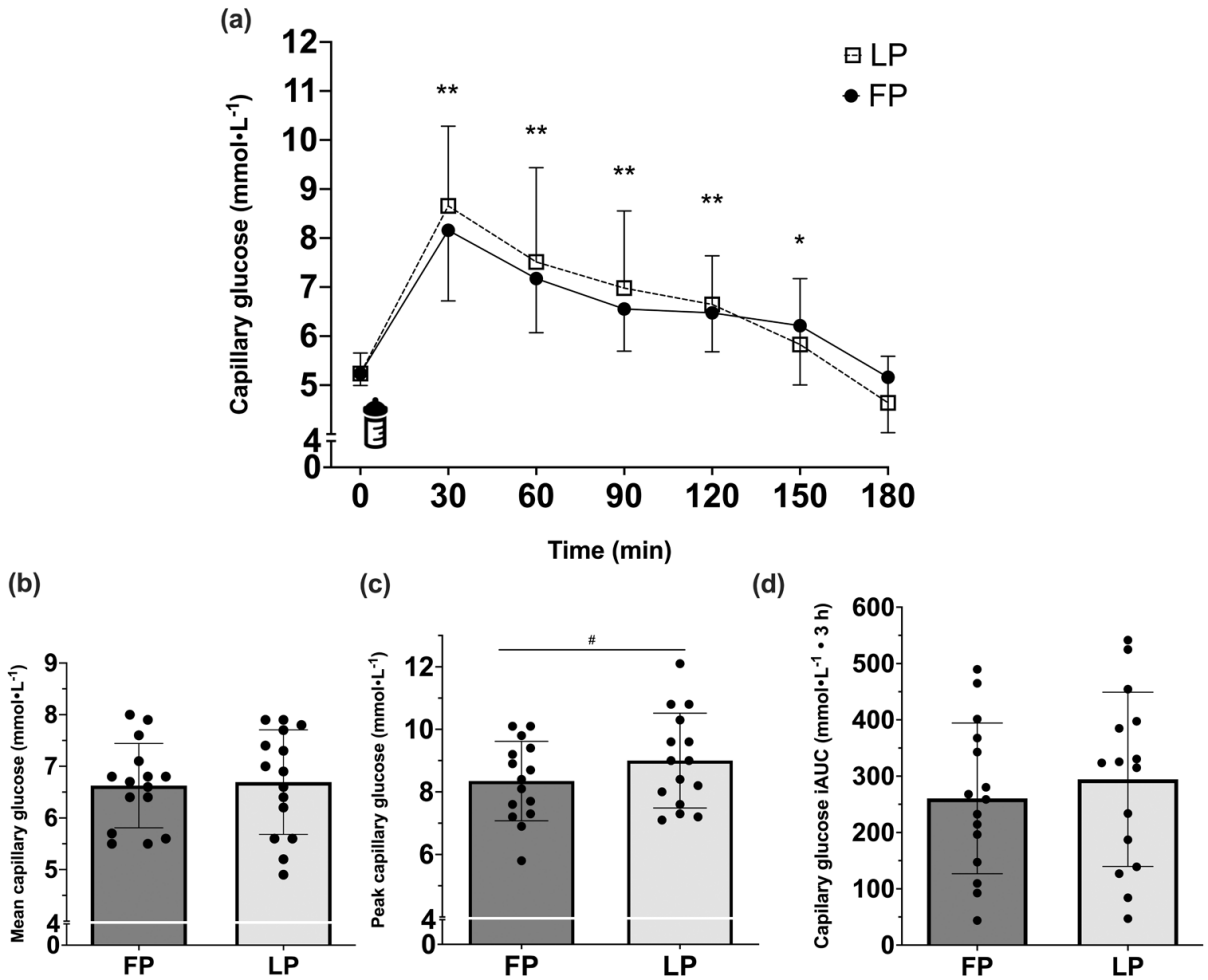
(a) Capillary glucose concentrations at rest in the overnight fasted state and in response to consumption of a 75 g glucose beverage in FP and LP. Glucose beverage (bottle icon) was consumed after fasted measurement at time “0”. **p < 0.0001, main effect of time (different than fasted), *p < 0.05, main effect of time (different than fasted). Capillary glucose mean (b), peak (c), and incremental area under the curve (iAUC; d). ^#^ *p* < 0.05, FP < LP. All values are expressed as means ± SD.

There was no interaction between menstrual cycle phase and time (*p* = 0.09, $\eta_{p}^{2}$ = 0.14, Figure 2a) or difference between menstrual cycle phases (main effect of phase, *p* = 0.64, $\eta_{p}^{2}$ = 0.02, Figure 2a) for blood glucose concentrations at rest and postprandially during the OGTT. However, there was a main effect of time (*p* < 0.001, $\eta_{p}^{2}$ = 0.71, Figure 2a) such that blood glucose concentration was elevated above fasting until 150 min postprandial (*p* = <0.0001–0.0387, *d* = 0.96–3.19). The 3 h postprandial glucose mean (Figure 2b) and iAUC (Figure 2d) were not different between menstrual cycle phases (*p* = 0.34–0.63, *d =* 0.10–0.23), however the 3 h postprandial glucose peak was higher in LP compared to FP (9.0 ± 1.5 vs. 8.3 ± 1.3 mmol/L, *p* = 0.04, *d* = 0.47, Figure 2c).

### 3.3 Respiratory gas exchange variables measured via indirect calorimetry

There were no significant interactions between menstrual cycle phase and time for any of the gas exchange variables shown in Table 2. However, there was a main effect of menstrual cycle phase for VO_2_, VCO_2_ and VE, with higher values observed in LP compared to FP (*p* = 0.003–0.02, $\eta_{p}^{2}$ = 0.38–0.51, Table 2).

**TABLE 2:** Gas exchange measures during rest when fasted, 60‐, 120‐, and 180‐min postprandial.

Variable (*n* = 14)	Time	FP	LP	Statistics		
				Time	Phase	Time × phase
RER	Fasted	0.76 ± 0.05	0.78 ± 0.05	*p* < 0.001 $\eta_{p}^{2}$ = 0.61	*p* = 0.12 $\eta_{p}^{2}$ = 0.18	*p* = 0.85 $\eta_{p}^{2}$ = 0.00
	60 min	0.83 ± 0.06	0.85 ± 0.07			
	120 min	0.82 ± 0.06	0.85 ± 0.06			
	180 min	0.82 ± 0.06	0.84 ± 0.07			
	Mean	0.81 ± 0.05	0.83 ± 0.05			
CHO oxidation (g/min)	Fasted	0.06 ± 0.03	0.07 ± 0.04	*p* < 0.001 $\eta_{p}^{2}$ = 0.54	*p* = 0.019 $\eta_{p}^{2}$ = 0.66	*p* = 0.48 $\eta_{p}^{2}$ = 0.07
	60 min	0.13 ± 0.07	0.17 ± 0.06			
	120 min	0.11 ± 0.07	0.15 ± 0.05			
	180 min	0.10 ± 0.05	0.13 ± 0.06			
	Mean	0.10 ± 0.05	0.13 ± 0.04			
Fat oxidation (g/min)	Fasted	0.08 ± 0.02	0.09 ± 0.03	*p* = 0.006 $\eta_{p}^{2}$ = 0.34	*p* = 0.36 $\eta_{p}^{2}$ = 0.08	*p* = 0.22 $\eta_{p}^{2}$ = 0.14
	60 min	0.06 ± 0.02	0.05 ± 0.03			
	120 min	0.08 ± 0.05	0.06 ± 0.03			
	180 min	0.07 ± 0.03	0.06 ± 0.04			
	Mean	0.07 ± 0.03	0.07 ± 0.03			
VO_2_ (L/min)	Fasted	0.20 ± 0.04	0.23 ± 0.03	*p* = 0.083 $\eta_{p}^{2}$ = 0.16	*p* = 0.012 $\eta_{p}^{2}$ = 0.38	*p* = 0.142 $\eta_{p}^{2}$ = 0.13
	60 min	0.22 ± 0.03	0.23 ± 0.04			
	120 min	0.22 ± 0.04	0.23 ± 0.02			
	180 min	0.21 ± 0.04	0.22 ± 0.03			
	Mean	0.21 ± 0.03	0.23 ± 0.03^a^			
VCO_2_ (L/min)	Fasted	0.16 ± 0.03	0.18 ± 0.02	*p* = 0.071 $\eta_{p}^{2}$ = 0.19	*p* = 0.016 $\eta_{p}^{2}$ = 0.38	*p* = 0.388 $\eta_{p}^{2}$ = 0.05
	60 min	0.18 ± 0.03	0.20 ± 0.03			
	120 min	0.18 ± 0.03	0.19 ± 0.02			
	180 min	0.17 ± 0.02	0.19 ± 0.02			
	Mean	0.17 ± 0.03	0.19 ± 0.02^a^			
FeO_2_ (%)	Fasted	17.70 ± 0.27	17.71 ± 0.31	*p* = 0.093 $\eta_{p}^{2}$ = 0.16	*p* = 0.188 $\eta_{p}^{2}$ = 0.13	*p* = 0.337 $\eta_{p}^{2}$ = 0.08
	60 min	17.64 ± 0.30	17.79 ± 0.37			
	120 min	17.66 ± 0.25	17.78 ± 0.33			
	180 min	17.73 ± 0.27	17.86 ± 0.32			
	Mean	17.68 ± 0.25	17.78 ± 0.30			
FeCO_2_ (%)	Fasted	2.63 ± 0.19	2.67 ± 0.29	*p* < 0.001 $\eta_{p}^{2}$ = 0.49	*p* = 0.759 $\eta_{p}^{2}$ = 0.01	*p* = 0.407 $\eta_{p}^{2}$ = 0.07
	60 min	2.86 ± 0.20^b^	2.80 ± 0.33^b^			
	120 min	2.84 ± 0.23^b^	2.80 ± 0.28^b^			
	180 min	2.76 ± 0.18	2.72 ± 0.27			
	Mean	2.77 ± 0.18	2.75 ± 0.27			
VT (L)	Fasted	0.48 ± 0.08	0.52 ± 0.09	*p* = 0.046 $\eta_{p}^{2}$ = 0.20	*p* = 0.252 $\eta_{p}^{2}$ = 0.10	*p* = 0.631 $\eta_{p}^{2}$ = 0.04
	60 min	0.51 ± 0.10	0.54 ± 0.12			
	120 min	0.50 ± 0.11	0.53 ± 0.05			
	180 min	0.48 ± 0.08	0.49 ± 0.06			
	Mean	0.50 ± 0.09	0.52 ± 0.07			
VE (L/min)	Fasted	7.35 ± 1.31	8.46 ± 1.40	*p* = 0.223 $\eta_{p}^{2}$ = 0.11	*p* = 0.003 $\eta_{p}^{2}$ = 0.51	*p* = 0.640 $\eta_{p}^{2}$ = 0.04
	60 min	8.00 ± 1.20	8.81 ± 1.84			
	120 min	7.93 ± 1.17	8.67 ± 1.09			
	180 min	7.66 ± 1.11	8.61 ± 1.31			
	Mean	7.74 ± 1.00	8.64 ± 1.24^a^			
Rf (breaths/min)	Fasted	15.71 ± 2.22	16.70 ± 3.39	*p* = 0.265 $\eta_{p}^{2}$ = 0.10	*p* = 0.127 $\eta_{p}^{2}$ = 0.17	*p* = 0.879 $\eta_{p}^{2}$ = 0.01
	60 min	15.98 ± 2.17	16.82 ± 3.25			
	120 min	15.36 ± 1.83	16.55 ± 2.11			
	180 min	16.30 ± 2.03	17.57 ± 2.91			
	Mean	15.84 ± 1.58	16.91 ± 2.57			

*Note*: Values are expressed as means ± SD. The mean over 180 min for each outcome is provided for both MC phases (FP and LP) below the time course data.

Abbreviations: FeCO_2_, fraction of expired carbon dioxide; FeO_2_, fraction of expired oxygen; RER, respiratory exchange ratio; Rf, breathing frequency; VCO_2_, volume of carbon dioxide; VE, minute ventilation; VO_2_, volume of oxygen; VO_2_, volume of oxygen; VT, tidal volume.

^**a**^ Higher in LP compared with FP (*p* < 0.05).

^b^ Higher than fasted (*p* < 0.05).

**FIGURE 3:**
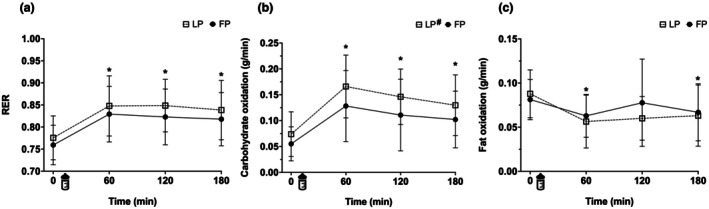
RER (a), rate of carbohydrate oxidation (b), and rate of fat oxidation (c) at rest in the overnight fasted state and in response to consumption of a 75 g glucose beverage in FP and LP. Glucose beverage (bottle icon) was consumed after fasted measurement at time “0”. ^#^Main effect of phase, LP > FP, *p* < 0.05. *Main effect of time compared to fasted (time “0”), *p* < 0.05. All values are expressed as means ± SD.

There were no significant interactions between menstrual cycle phase and time for FeCO_2_ (*p* = 0.407, $\eta_{p}^{2}$ = 0.07, Table 2) or RER (*p* = 0.85, $\eta_{p}^{2}$ = 0.00, Figure 3a), or main effects of menstrual phase for either (FeCO_2_: *p* = 0.76, $\eta_{p}^{2}$ = 0.01, Table 2; RER: *p* = 0.12, $\eta_{p}^{2}$ = 0.18, Figure 3a). However, there was a significant main effect of time for both variables (FeCO_2_: *p* < 0.001, $\eta_{p}^{2}$ = 0.49, Table 2; RER: *p* < 0.001, $\eta_{p}^{2}$ = 0.61, Figure 3a), such that both were increased above fasting at all postprandial timepoints (*p* = <0.001–0.010, *d* = 0.49–1.43).

There was a main effect of time for VT (*p* = 0.046, $\eta_{p}^{2}$ = 0.20, Table 2); however, pairwise comparisons revealed no significant differences at any timepoint when adjusted for multiple comparisons (*p* = 0.05–1.0, *d* = 0.19–0.27). Lastly, there was no effect of menstrual cycle phase or time for FeO_2_ or Rf (*p* = 0.09–0.88, $\eta_{p}^{2}$ = 0.01–0.17, Table 2).

### 3.4 Calculated rates of carbohydrate and fat oxidation via indirect calorimetry

There were no significant interactions between menstrual cycle phase and time (*p* = 0.48, $\eta_{p}^{2}$ = 0.07, Figure 3b) for the rate of whole‐body carbohydrate oxidation. However, there was a main effect of menstrual cycle phase (*p* = 0.019, $\eta_{p}^{2}$ = 0.36, Figure 3b) such that the rate of carbohydrate oxidation was higher in LP compared to FP (0.13 ± 0.04 vs. 0.10 ± 0.0 g/min, *p* = 0.019, d = 0.66, Figure 3b) across all timepoints. In addition, there was a main effect of time (*p* < 0.001, $\eta_{p}^{2}$ = 0.54, Figure 3b) such that carbohydrate oxidation rate was increased above fasting at all postprandial timepoints in both trials (all *p =* <0.001–0.003, d = 1.21–1.83).

There were no significant interactions between menstrual cycle phase and time (*p* = 0.22, $\eta_{p}^{2}$ = 0.14, Figure 3c) or main effect of menstrual cycle phase (*p* = 0.36, $\eta_{p}^{2}$ = 0.08, Figure 3c) for the rate of whole‐body fat oxidation. However, there was a main effect of time (*p* = 0.006, $\eta_{p}^{2}$ = 0.34, Figure 3c) such that rates of fat oxidation were decreased below fasting at 1 h (*p* < 0.001, d = 1.11) and 3 h (*p* = 0.003, d = 0.70), but not 2 h (*p* = 0.31, d = 0.55) postprandial.

### 3.5 Exogenous glucose oxidation via [U‐ ^13^C_6_ ] D‐glucose tracer

**FIGURE 4:**
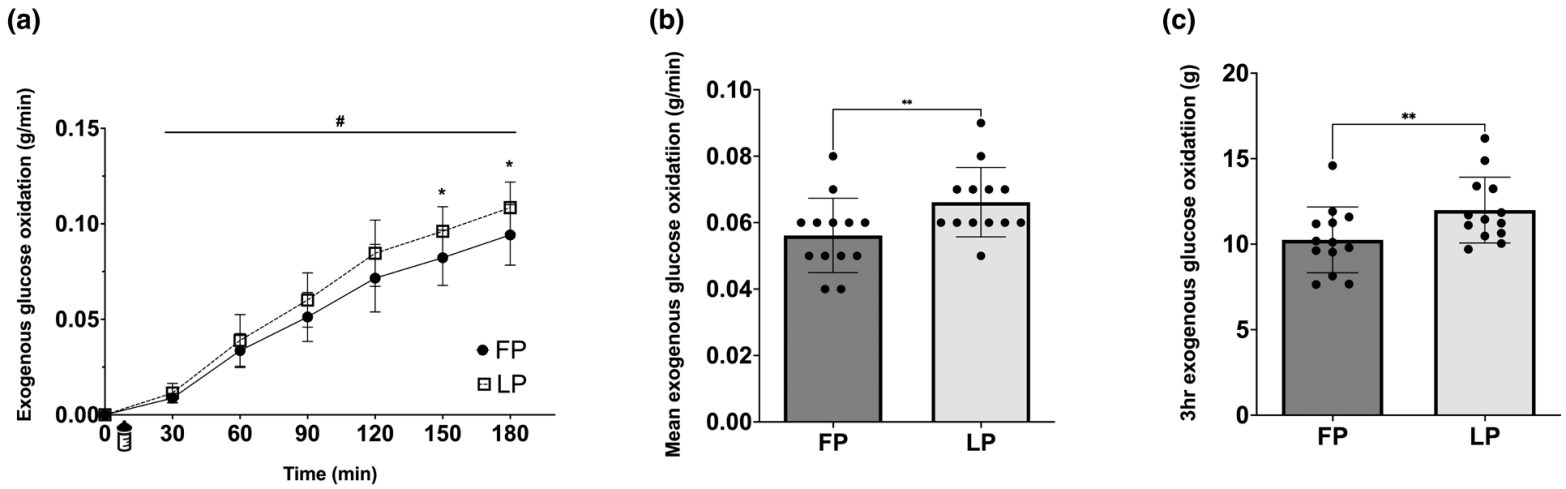
Exogenous postprandial glucose oxidation (a), mean exogenous glucose oxidation over 3 h (b), and total exogenous glucose oxidation over 3 h (c) in response to consumption of a 75 g glucose beverage in FP and LP. Glucose beverage (bottle icon) was consumed after fasted measurement at time “0”. Main effect of time compared to fasted (time “0”), ^#^ *p* < 0.001. *p < 0.05, LP > FP; **p < 0.01, LP>FP. All values are expressed as means ± SD.

There was an interaction (phase × time; *p* < 0.001, $\eta_{p}^{2}$ = 0.33) for rates of exogenous glucose oxidation. Post hoc analysis revealed that rates of exogenous glucose oxidation were higher in LP compared to FP at 150 min (0.10 ± 0.01 vs. 0.08 ± 0.01 g/min, *p* = 0.02, *d* = 1.01) and 180 min (0.11 ± 0.01 vs. 0.09 ± 0.02 g/min, *p* = 0.02, *d* = 0.98) following glucose ingestion (Figure 4a). Additionally, rates of exogenous glucose oxidation were elevated above fasting at all postprandial timepoints in both trials (*p* < 0.001, *d* = 4.9–10.8). The 3 h mean postprandial exogenous glucose oxidation rate was higher in LP vs. FP (0.067 ± 0.011 vs. 0.057 ± 0.011 g/min, *p* = 0.002, *d* = 0.92, Figure 4b). The total exogenous glucose oxidized over the 3 h postprandial period was higher in LP vs. FP (12.0 ± 1.9 vs. 10.3 ± 1.9 g, *p* = 0.002, *d* = 0.91, Figure 4c). To address the possibility that differences in calculated exogenous glucose oxidation were driven by phase‐related differences in VCO_2_, we additionally analyzed breath ^13^CO_2_ enrichment (APE), corrected for baseline enrichment, independent of VCO_2_. The area under the curve (AUC 0–3 h) for breath ^13^CO₂ APE was significantly higher in LP compared with FP (3.28 ± 0.54 vs. 3.04% ± 0.50% × 3 h, *p* = 0.046, *d* = 0.46).

### 3.6 Lumen L%CO_2_

There was no interaction between menstrual cycle phase and time (*p* = 0.31, $\eta_{p}^{2}$ = 0.08), or main effect of menstrual cycle phase (*p* = 0.84, $\eta_{p}^{2}$ = 0.00). However, there was a main effect of time (*p* < 0.001, $\eta_{p}^{2}$ = 0.22) such that %CO_2_ was increased above fasting at all postprandial timepoints. There was also no difference in fasting %CO_2_ (4.26 ± 0.35 vs. 4.25 ± 0.21%, *p* = 0.87, *d* = 0.05) or 3 h %CO_2_ mean (4.39 ± 0.22 vs. 4.41 ± 0.29%, *p* = 0.75, *d* = 0.08) between LP and FP.

## 4 DISCUSSION

The present study was designed to determine whether menstrual cycle phase influences carbohydrate metabolism in response to glucose ingestion among healthy eumenorrheic females. We observed that peak capillary blood glucose concentration was higher in the LP compared to the FP following a 75 g OGTT; however, there was no difference in 3 h glucose mean or iAUC across phases. Furthermore, there was an increase in the carbohydrate oxidation rate during the OGTT in the LP compared to FP as measured via indirect calorimetry and a [U‐^13^C_6_] glucose tracer, which was contrary to our initial hypothesis. These findings demonstrate that menstrual cycle phase influences indices of carbohydrate metabolism in response to oral glucose intake among healthy young females.

In the present study, we observed higher peak capillary blood glucose concentrations during a 75 g OGTT in LP compared to FP, although no differences were observed in 3 h postprandial glucose mean or iAUC between phases. Our finding of elevated peak glucose in the LP relative to FP aligns with previous reports using a 50 g OGTT in healthy eumenorrheic females (Brennan et al., 2009). In contrast to our results, some studies have observed no effect of menstrual cycle phase on postprandial blood glucose concentrations following an OGTT in healthy eumenorrheic females (Bonora et al., 1987; Cudworth & Veevers, 1975). Differences across studies may partly reflect the various methods used to characterize menstrual cycle phase. In our study, phase was verified using both calendar tracking and ovulation testing, whereas some previous work relied on calendar‐based estimation alone (Bonora et al., 1987; Cudworth & Veevers, 1975). Notably, menstrual cycle length in our participants ranged from 26 to 35 days, and ovulation occurred between days 11 and 17, illustrating the potential for misclassification when phase is identified purely by calendar tracking.

Mechanisms underlying the differences in peak glucose between phases were not evaluated in the present study but may be related to phase‐specific differences in gastric emptying and/or insulin sensitivity. Blood glucose concentration 30 min after glucose ingestion is positively correlated with the rate of gastric emptying (Horowitz et al., 1993), which has been reported to be faster in the LP (Brennan et al., 2009). Alternatively, it is possible that elevations in blood glucose during the LP may arise due to lower insulin sensitivity previously reported in this phase (Pulido & Salazar, 1999; Valdes & Elkind‐Hirsch, 1991), which could be related to increased progesterone concentration during the LP (Kumagai et al., 1993). However, given that most metrics of postprandial glycemia did not differ between phases in our study, we interpret the transiently higher glucose excursion as a normal physiological variation rather than a sign of glucose intolerance in the luteal phase.

In the present study, we observed that the rate of carbohydrate oxidation in both the fasted and postprandial state was elevated in LP compared to FP, as measured by indirect calorimetry. VO_2_ was also modestly elevated in LP, reflecting higher energy expenditure consistent with other reports (Benton et al., 2020). Using fasted VO_2_ values and assuming 5 kcal per 1 L O_2_ consumed, we calculated that daily energy expenditure was approximately 193 kcal/day higher in the LP than in the FP (*p* = 0.0012). This elevation in energy expenditure within the LP is comparable to that reported elsewhere (Benton et al., 2020) and may reflect the hyperthermic effects of high progesterone levels during the mid‐luteal phase, which have been proposed to increase resting energy expenditure in naturally cycling females (Solomon et al., 1982). Thus, the greater carbohydrate oxidation observed in the LP likely reflects increased overall energy expenditure rather than a change in substrate use, as fat oxidation and RER were not significantly different between phases.

Few studies have evaluated the influence of menstrual cycle phase on rested postprandial substrate oxidation, limiting direct comparisons with our findings. By contrast, studies conducted during fasted exercise have reported lower carbohydrate oxidation in LP compared to FP (Hackney et al., 1994; Zderic et al., 2001), although no difference between phases has also been observed (Kanaley et al., 1992). The seeming discrepancy between these findings and our results suggests that higher carbohydrate oxidation in the LP may be a result of differences in energy demand and/or fuel selection between rested and exercise conditions. At rest, and particularly in the postprandial state, energy requirements are primarily met by blood‐borne fuels such as glucose, whereas the heightened energy demands during exercise shift reliance toward fuel sources within skeletal muscle (van Loon et al., 2001). These distinctions limit the ability to extrapolate metabolic responses during exercise to resting conditions, and vice versa.

Consistent with our findings, Williams and colleagues (2023) found elevated carbohydrate oxidation in the LP compared to FP during fasted supine rest (0.09 vs. 0.07 g/min), although multiple comparison groups in this study (e.g., three oral contraceptive types) may have reduced statistical power and obscured statistical significance (Williams et al., 2023). Despite these observations, a recent systematic review and meta‐analysis reported no significant differences in substrate oxidation between FP and LP in the fasted state at rest (D'Souza et al., 2023), although the authors reported that the studies included provide a low certainty of evidence. Importantly, this analysis primarily relied on RER as an index of substrate use. While RER reflects the relative contribution of carbohydrate versus fat to total energy expenditure (i.e., fuel mix), it does not quantify absolute substrate oxidation rates. As such, increases in carbohydrate oxidation can occur without detectable changes in RER if total energy expenditure is concurrently elevated, as was observed in our study, where RER was unchanged despite higher carbohydrate oxidation in the LP.

Consistent with others (Hayashi et al., 2012; Hazlett & Edgell, 2018; Takano, 1984; Williams et al., 2023), minute ventilation was ~1 L/min higher in LP compared to FP in the present study. Increased ventilation observed in LP has been attributed to elevated progesterone concentrations, which enhance chemoreceptor sensitivity and stimulate respiratory drive (Jensen et al., 2005; Zwillich et al., 1978). As minute ventilation directly influences the calculation of carbohydrate oxidation rate (formula provided within methods) we cannot exclude the possibility that the difference in respiration contributed to the differences in whole‐body carbohydrate oxidation observed in our study. However, the proportional increases in VO_2_ and VCO_2_ suggest that the greater carbohydrate oxidation reflects a metabolic shift rather than a ventilatory artifact. The parallel rise in minute ventilation may instead represent a normal luteal‐phase increase in respiratory drive associated with progesterone.

To quantify exogenous glucose oxidation during the postprandial period, we enriched the OGTT beverage with 0.1% [U‐^13^C_6_] D‐glucose. While this approach has been used to compare individuals with normoglycemia and type 2 diabetes (Dillon et al., 2009; Ghosh et al., 2015), it has not previously been applied to investigate metabolic responses across the menstrual cycle. We found that exogenous glucose oxidation was higher in the LP compared to the FP, aligning with the phase‐specific increase in whole‐body carbohydrate oxidation rates estimated by indirect calorimetry. The greater exogenous glucose oxidation in the LP appeared to reflect contributions from both metabolic and respiratory factors. Specifically, breath ^13^CO_2_ enrichment (APE), assessed independent of VCO_2_, was higher in the LP, as indicated by the greater AUC for breath ^13^CO_2_. This greater tracer enrichment was further augmented when combined with higher VCO_2_ during the LP, resulting in congruently higher calculated rates of exogenous glucose oxidation.

We also employed a portable Lumen breath analyzer to assess L%CO_2_ as an index of fat oxidation and found no significant effect of menstrual cycle phase. The findings are consistent with that of the fat oxidation rate determined via indirect calorimetry, where we observed no significant phase‐based differences in fat oxidation rate. In contrast to our findings, a recent retrospective cohort study involving 1191 eumenorrheic, naturally cycling females observed lower L%CO_2_ (indicative of higher fat oxidation) in the LP compared to FP under free‐living conditions (Cramer et al., 2024). This finding aligns with prior reports of a negative correlation between arterial PCO_2_ and circulating progesterone concentrations (Jensen et al., 2005). The lack of difference in L%CO_2_ between menstrual cycle phases in our study may be attributed to our lower sample size and/or differences in nutritional state compared to previous research. Notably, our protocol assessed L%CO_2_ during the immediate postprandial period, whereas Cramer et al. (2024) conducted a retrospective analysis under free‐living conditions, encompassing a broader range of metabolic states (e.g., fasted, fed, post‐exercise, etc.). It is plausible that menstrual cycle differences in fat metabolism observed across an entire day may not be apparent in the immediate postprandial period following and oral glucose load. Additionally, given that a recent meta‐analysis suggests that healthy, eumenorrheic pre‐menopausal females consume an additional ~170 kcal/day in the LP compared to FP (Tucker et al., 2024), it is possible that differences in free‐living energy intake across the menstrual cycle contributes to the observed differences in substrate oxidation measured outside of a laboratory setting.

Our study has a number of strengths, including the use of multiple methodological techniques to assess substrate oxidation in humans. In addition, our incorporation of ovulation testing for menstrual cycle phase verification enhances the accuracy of phase classification and addresses some of the methodological limitations observed in previous studies. Nonetheless, there are also limitations to consider. Venous blood samples were not obtained in the present study and therefore we were unable to measure relevant glycemic hormones such as insulin and GLP‐1, which limits understanding of the mechanisms contributing to the phase‐based differences in peak glucose. Similarly, we were unable to measure serum estradiol and progesterone concentrations. Best practices for menstrual phase verification recommend a three‐step approach of calendar‐based tracking, ovulation testing, and confirmation with blood hormone measurements (Elliott‐Sale et al., 2021). Although omission of blood sampling is not uncommon in studies evaluating substrate oxidation using non‐invasive methods (Williams et al., 2023), incorporating these measurements in future studies would strengthen conclusions. Additionally, although the sample size was based on a power calculation for our primary outcome (blood glucose), significant differences in secondary outcomes, such as RER, may have gone undetected due to lack of power. Indeed, a retrospective power calculation suggests that *n* = 26 would be required to detect a significant difference in RER between FP and LP (0.81 vs. 0.83). Finally, in the present study we aimed to directly compare FP and LP within a single menstrual cycle. As a result, participants completed the first metabolic trial in the FP and the second in the LP, which introduces the potential for an order effect on our outcomes which should be considered.

## 5 CONCLUSION

To conclude, our study demonstrates that peak capillary blood glucose concentration and rates of whole‐body and exogenous carbohydrate oxidation in response to a 75 g oral glucose load are higher in the LP compared to FP in healthy, eumenorrheic women. These differences appear to reflect normal physiological variation across the menstrual cycle rather than any indication of metabolic dysfunction, as overall glycemic responses were well within the expected range. Nonetheless, these phase‐related differences may be important to consider when designing and interpreting metabolic studies in females. Future work is needed to determine whether similar patterns occur in females at elevated risk for metabolic disease or following the consumption of mixed‐macronutrient meals more representative of typical Western dietary patterns.

## AUTHOR CONTRIBUTIONS

**Alexa Govette:** Conceptualization; data curation; formal analysis; investigation; validation; visualization. **Daniel W. D. West:** Data curation; formal analysis; investigation; resources. **Madeleine Pettit:** Data curation; formal analysis; investigation. **Eric Antonen:** Data curation; formal analysis; investigation. **Anessa Koussiouris:** Data curation; formal analysis; investigation. **Daniel R. Moore:** Data curation; funding acquisition; investigation; resources. **Jenna B. Gillen:** Conceptualization; data curation; formal analysis; funding acquisition; investigation; methodology; project administration; resources; supervision.

## FUNDING INFORMATION

This study was supported by a Natural Sciences and Engineering Research Council (NSERC) Discovery grant (RGPIN‐2020‐05779) to JBG.

## CONFLICT OF INTEREST STATEMENT

No conflicts of interest, financial or otherwise, are declared by the authors.

## ETHICS STATEMENT

This study was conducted according to the guidelines laid down in the Declaration of Helsinki and all procedures involving human participants were approved by the University of Toronto Health Sciences Research Ethics Board (#40004). Written informed consent was obtained from all participants.

## Data Availability

The data supporting the findings of this study are included within the text, figures, and tables of the present article.
